# Uncalibrated pulse power analysis fails to reliably measure cardiac output in patients undergoing coronary artery bypass surgery

**DOI:** 10.1186/cc10065

**Published:** 2011-02-28

**Authors:** Ole Broch, Jochen Renner, Jan Höcker, Matthias Gruenewald, Patrick Meybohm, Jan Schöttler, Markus Steinfath, Berthold Bein

**Affiliations:** 1 Department of Anaesthesiology and Intensive Care Medicine, University Hospital Schleswig-Holstein, Campus Kiel, Schwanenweg 21, 24105 Kiel, Germany; 2 Department of Cardiothoracic and Vascular Surgery, University Hospital Schleswig-Holstein, Campus Kiel, Arnold-Heller-Straße 7, 24105 Kiel, Germany

## Abstract

**Introduction:**

Uncalibrated arterial pulse power analysis has been recently introduced for continuous monitoring of cardiac index (CI). The aim of the present study was to compare the accuracy of arterial pulse power analysis with intermittent transpulmonary thermodilution (TPTD) before and after cardiopulmonary bypass (CPB).

**Methods:**

Forty-two patients scheduled for elective coronary surgery were studied after induction of anaesthesia, before and after CPB respectively. Each patient was monitored with the pulse contour cardiac output (PiCCO) system, a central venous line and the recently introduced LiDCO monitoring system. Haemodynamic variables included measurement of CI derived by transpulmonary thermodilution (CI_TPTD_) or CI derived by pulse power analysis (CI_PP_), before and after calibration (CI_PPnon-cal._, CI_PPcal._). Percentage changes of CI (ΔCI_TPTD_, ΔCI_PPnon-cal./PPcal._) were calculated to analyse directional changes.

**Results:**

Before CPB there was no significant correlation between CI_PPnon-cal. _and CI_TPTD _(r^2 ^= 0.04, *P *= 0.08) with a percentage error (PE) of 86%. Higher mean arterial pressure (MAP) values were significantly correlated with higher CI_PPnon-cal. _(r^2 ^= 0.26, *P *< 0.0001). After CPB, CI_PPcal. _revealed a significant correlation compared with CI_TPTD _(r^2 ^= 0.77, *P *< 0.0001) with PE of 28%. Changes in CI_PPcal. _(ΔCI_PPcal._) showed a correlation with changes in CI_TPTD _(ΔCI_TPTD_) only after CPB (r^2 ^= 0.52, *P *= 0.005).

**Conclusions:**

Uncalibrated pulse power analysis was significantly influenced by MAP and was not able to reliably measure CI compared with TPTD. Calibration improved accuracy, but pulse power analysis was still not consistently interchangeable with TPTD. Only calibrated pulse power analysis was able to reliably track haemodynamic changes and trends.

## Introduction

Measuring left ventricular stroke volume and cardiac index (CI) have gained increasing impact regarding perioperative monitoring of critically ill patients either in the operating theatre or on the intensive care unit. Goal-directed perioperative optimization of left ventricular stroke volume and CI have a positive impact on the morbidity and the length of stay on the intensive care unit [1-4]. Measurement of CI with the pulmonary artery catheter (PAC) is still widely used and often considered as a kind of "gold standard" in different clinical settings [5,6]. However, several studies showed that pulmonary artery catheterization has clinical limitations and bares the potential risk for severe complications [7-9]. In this context, interest has focused on less invasive techniques which are based for example on transpulmonary thermodilution (TPTD) or arterial waveform analysis [6,10,11]. Alternative methods of haemodynamic monitoring for estimating CI such as transpulmonary thermodilution differ from pulmonary artery thermodilution and are theoretically more sensitive to thermal blood loss and changes such as recirculation and forward-backward movement, especially in the presence of left-sided valvular insufficiencies [12]. It has been repeatedly shown, however, that pulmonary artery thermodilution and transpulmonary thermodilution are interchangeable in different patient populations and during different surgical procedures [6,13-15].

The recently introduced LiDCO monitoring system (LiDCO_*Rapid*_; LiDCO Group Ltd, London, UK) consists of an arterial pressure waveform analysis that provides beat-to-beat measurement of CI by analysis of the arterial blood pressure tracing. The underlying pulse power algorithm (PulseCO) originally was introduced as an algorithm requiring calibration by lithium indicator dilution to determine the individual vascular compliance and has been evaluated in different clinical scenarios [16,17]. Using a nomogram to assess the patient specific aortic compliance, the new software version estimates stroke volume without the need for calibration. Furthermore, this device offers the possibility of calibration by a reference technique. Based on these updates, LiDCO_*Rapid *_only requires a standard radial arterial line and is claimed to mirror CI or trends of CI reliably. However, calculation of cardiac index by arterial pressure waveform analysis could be influenced by several confounders, like changes in vascular tone or vasoactive drugs [18,19]. Specifically, it has been shown that methods based on arterial waveform analysis are prone to failure after cardiopulmonary bypass (CPB), when major changes in vascular resistance are likely to occur [15].

Therefore, the aim of the present study was to investigate the accuracy of uncalibrated and calibrated pulse power analysis (CI_PPnon-cal._, CI_PPcal._) with respect to simultaneous measurements and the ability to track haemodynamic changes (ΔCI_TPTD_, ΔCI_PPnon-cal./cal._), both before and after CPB.

## Materials and methods

Approval from our institutional ethics committee (Christian Albrecht University Kiel) was obtained and all patients gave informed consent for participation in the study.

Forty-two patients undergoing elective coronary artery bypass grafting (CABG) were studied after induction of general anaesthesia. Inclusion criteria were as follows: patients >18 years of age with a left ventricular ejection fraction ≥0.5. Patients with emergency procedures, haemodynamic instability requiring inotropic and/or vasoactive pharmacologic support, intracardiac shunts, severe aortic-, tricuspid- or mitral stenosis or insufficiency, and patients on an intra-aortic balloon pump were all excluded from the study.

### Instrumentation and protocol

All patients were pre-medicated with midazolam 0.1 mg·kg^-1 ^orally 30 minutes before induction of anaesthesia. Routine monitoring was established including non-invasive blood pressure (NIBP), peripheral oxygen saturation (SpO_2_) and heart rate (HR) by electrocardiogram (ECG; S/5, GE Healthcare, Helsinki, Finland). Subsequently patients received a peripheral venous access and a radial arterial pressure catheter. The LiDCO_*Rapid *_monitor was connected to the S/5 monitor and started after input of patient specific data according to the manufacturer's instructions. After induction of anaesthesia with sufentanil (0.5 μg·kg^-1^) and propofol (1.5 mg·kg^-1^), orotracheal intubation was facilitated with rocuronium (0.6 mg·kg^-1^). Anaesthesia was maintained with sufentanil (1 μg·kg^-1^·h^-1^) and propofol (3 mg·kg^-1^·h^-1^). Patients were ventilated with an oxygen/air mixture using a tidal volume of 8 ml·kg^-1 ^and positive end-expiratory pressure was set at 5 cmH₂O. A central venous catheter and a thermodilution catheter (Pulsion Medical Systems, Munich, Germany) were introduced in the right internal jugular vein, respectively in the femoral artery and the thermodilution catheter was connected to the PiCCO monitor (PiCCOplus, software version 6.0; Pulsion Medical Systems, Munich, Germany).

### Data collection

**Figure 1:**
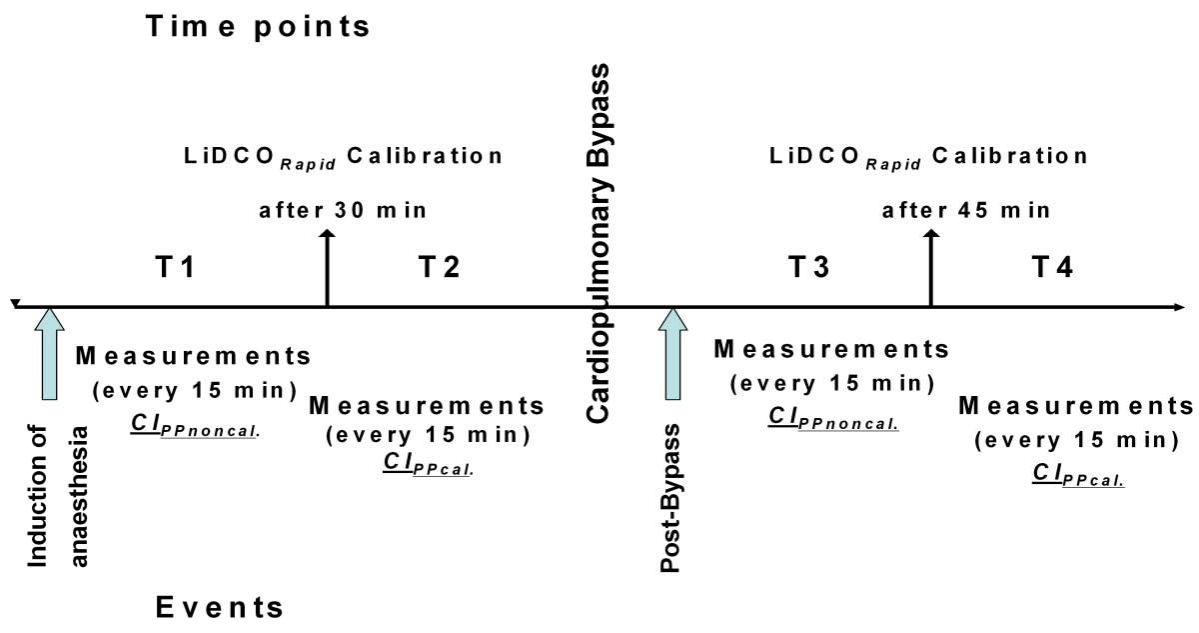
**Study design**. T1: data collection after induction of anaesthesia until calibration (CI_PPnon-cal._). T2: after calibration until cardiopulmonary bypass (CI_PPcal._). T3: after cardiopulmonary bypass until calibration (CI_PPnon-cal._). T4: after calibration until discharge to the intensive care unit (CI_PPcal._).

Measurements of CI_TPTD _were performed every 15 minutes by injecting 15 ml ice cold saline (≤8°C) through the central venous line. Injections were repeated at least three times and randomly assigned to the respiratory cycle. In case of a difference with respect to the preceding CI_TPTD _measurement of ≥15%, the value obtained was discarded and the measurement repeated. Measurements of CI_PP _were performed by plotting 10 numerical values over a period of one minute, excluding variations ≥15% and determining the mean value. Mean arterial pressure and CVP were also recorded every 15 minutes. Values of CI_PPnon-cal._, and CI_PPcal. _were collected during a one minute period and averaged. After induction of anaesthesia, haemodynamic variables including CI_TPTD _and CI_PPnon-cal. _were recorded every 15 minutes up to 30 minutes (T1), which means two pairs of measurements. After 30 minutes, calibration of pulse power analysis (CI_PPcal._) was performed and measurements were recorded until the beginning of CPB (T2), which differed from patient to patient yielding different numbers of measurements in this time period. Measurements were restarted 15 minutes after weaning from CPB. Subsequently, measurements of CI_TPTD _and CI_PPnon-cal. _were obtained up to 45 minutes (T3), yielding three pairs of measurements. After 45 minutes, re-calibration of pulse power analysis (CI_PPcal._) was carried out and haemodynamic variables were recorded until the patient was discharged to the intensive care unit (T4), again yielding a different number of measurement pairs in individual patients. Two patients were discharged to the intensive care unit 45 minutes after CPB, therefore, CI_PPcal. _measurements were not available from these patients. The study design is displayed in Figure 1.

### Statistical analysis

All data are given as mean ± SD. Statistical comparisons were performed using commercially available statistics software (GraphPad Prism 5, GraphPad Software Inc., San Diego, CA, USA, Software R, R Foundation for Statistical Computing, Vienna, Austria and PASS Version 11, NCSS, LLC. Kaysville, UT, USA). To demonstrate the relationship between sample size and the width of the confidence interval of the estimated variable, we calculated the width of the 95% confidence interval of the limits of agreement (0.52 standard deviations of the bias). To describe the agreement between CI_TPTD, _CI_PPnon-cal. _and CI_PPcal._, Bland-Altman plots were calculated for each time period (T1 to T4) before and after CPB. Percentage error was calculated as described by Critchley and colleagues, using the limits of agreement (2SD) of the bias divided by the mean CI values from CI_TPTD_, CI_PPnon-cal. _and CI_PPcal._. Bland-Altman plots were also performed for haemodynamic trends (ΔCI_TPTD, _ΔCI_PPnon-cal. _and ΔCI_PPcal._) before and after CPB. ΔCI_TPTD _<15% were excluded from analysis as recommended by Critchley and co-workers [20]. To describe the discriminative power of ΔCI_PPnon-cal. _and ΔCI_PPcal. _predicting true changes in CI_TPTD _(>15%) ROC analysis was performed. Post hoc power of ROC analysis was calculated with PASS software. Dependent upon the number of subjects enrolled at each time point (T1 to T4) the difference with respect to AUC between the null hypothesis (AUC = 0.50) and the alternative hypothesis (AUC of ΔCI_PPnon-cal. _and ΔCI_PPcal. _>0.50) that could be detected ranged from 0.28 to 0.32 for an α = 0.05 and a β = 0.20. An unpaired sample t-test was used to analyse significant differences of mean arterial pressure related to the periods of measurement.

## Results

Data from all 42 patients, 31 males and 11 females, were included in the final analysis. Ages ranged between 41 to 78 years, with a mean age of 63 ± 5 and a mean body mass index of 27.4 ± 4.9 kg/m^2^. Mean left ventricular ejection fraction was 0.58 ± 0.04%. A total of 430 data pairs (T1: 84, T2: 164, T3: 123, T4: 59) were obtained during the study period. An unpaired t-test showed a significant difference (*P *< 0.05) between MAP values before (T1, T2) and after cardiopulmonary bypass (T3, T4). Haemodynamic and respiratory variables are shown in Table 1.

**Table 1:** Haemodynamic and respiratory variables at different time points

		Pre - Bypass			Post - Bypass		
			
Variables	Time points Data pairs	T1 n = 84	T2 n = 164	*P*	T3 n = 123	T4 n = 59	*P*
**HR (minute**^**-1**^**)**		55 ± 2	56 ± 3	*P P *= 0.45	80 ± 3^§^	82 ± 2^§^	*P P *= 0.33
**MAP (mmHg)**		83 ± 17	76 ± 12	*P *<0.05	68 ± 7^§^	67 ± 5^§^	*P *= 0.98
**CVP (mmHg)**		10 ± 2	11 ± 2	*P *= 0.54	9 ± 1	11 ± 1	*P 0 *= 0.10
**Lung compliance (mL/cmH**_**2**_**O)**		51 ± 2	53 ± 1	*P *= 0.22	50 ± 2	49 ± 2	*P *= 0.67
**Tidal volume (mL)**		675 ± 75	686 ± 69	*P *= 0.15	700 ± 72	695 ± 70	*P *= 0.39
**SVRI (dynes**∙**s/cm**^**5**^**/m**^**2**^**)**		2,712 ± 68	2,096 ± 327	*P *<0.05	1,659 ± 141^§^	1 729 ± 138^§^	*P *= 0.11
**CI**_**PPnon-cal. **_**(L/minute/m**^**2**^**)**		2.5 ± 0.7			3.4 ± 0.2 *		
**CI**_**PPcal. **_**(L/minute/m**^**2**^**)**			2.6 ± 0.2			3.2 ± 0.1^#^	
**CI**_**TPTD **_**(L/minute/m**^**2**^**)**		2.3 ± 0.1	2.4 ± 0.1	*P *= 0.17	3.3 ± 0.2^§^	3.3 ± 0.2^§^	*P *= 0.55

HR, heart rate; MAP, mean arterial HR, heart.

CI_PPnon-cal._, cardiac index by uncalibrated pulse power analysis; CI_PPcal._, cardiac index by calibrated pulse power analysis; CI_TPTD_, cardiac index by transpulmonary thermodilution; CVP, central venous pressure; HR, heart rate; MAP, mean arterial pressure;, stroke volume index by transpulmonary thermodilution; SVRI, systemic vascular resistance index; SVI_TPTD. _Values are given as mean ± SD.

^§^*P *< 0.05 (vs. T1, T2), *P < 0.05 (vs. T1), ^# ^*P *< 0.05 (vs. T2).

**Figure 2:**
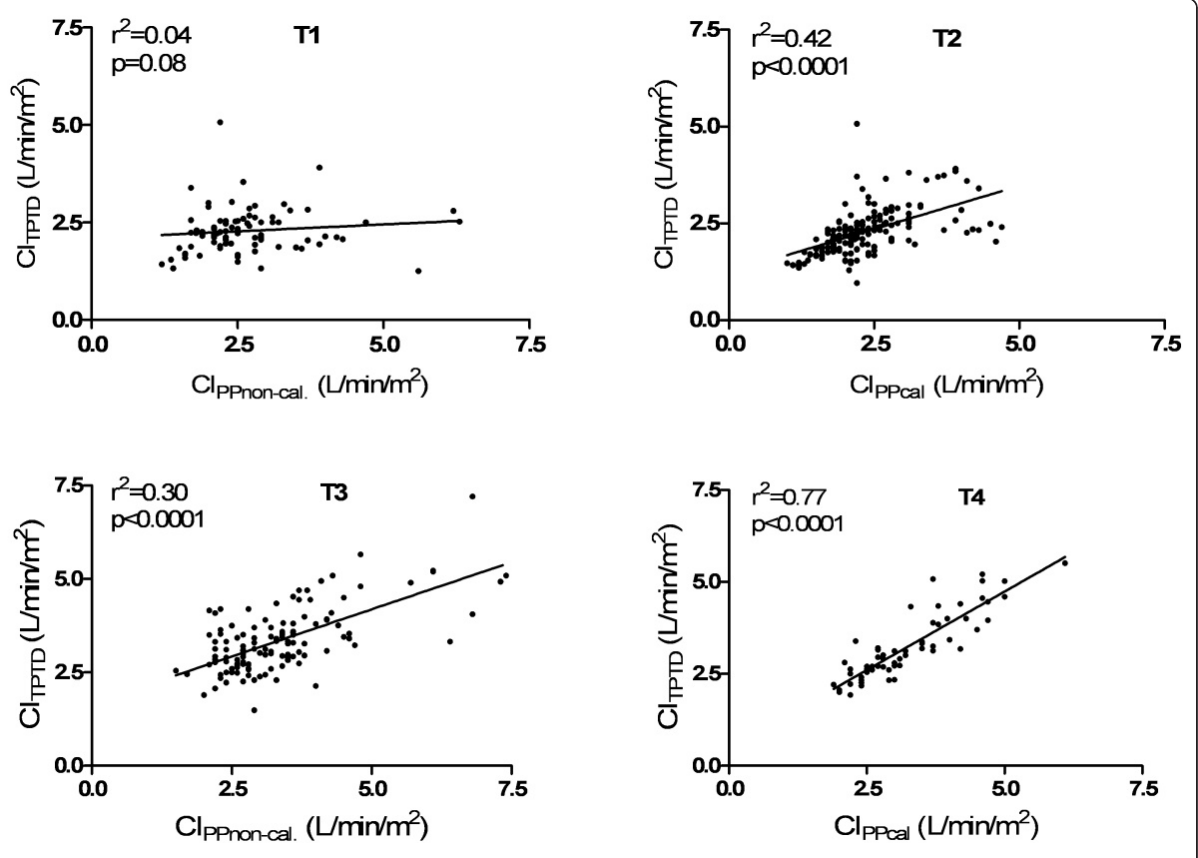
**Correlation of cardiac indices before (T1, T2) and after (T3, T4) cardiopulmonary bypass**.

There was no significant correlation between CI_PPnon-cal. _and CI_TPTD _(r^2 ^= 0.04, *P *= 0.08, n = 84) within the first 30 minutes (T1) after induction of anaesthesia (Figure 2). Bland-Altman analysis showed a mean bias of 0.36 L/minute/m^2 ^(95% limits of agreement (LOA): -1.73 to +2.46 L/minute/m^2^) with a percentage error (PE) of 86%. Bias, LOA and PE for each time period (T1 to T4) are summarized in Table 2. Correlation between CI_TPTD _and CI_PP _is shown in Figure 2. CI_PPcal. _(T2) revealed a significant correlation with CI_TPTD _(r^2 ^= 0.42, *P *< 0.0001, n = 164) and Bland-Altman analysis showed a mean bias of 0.075 L/minute^1^/m^2 ^(LOA: -1.19 to + 1.34 L/minute/m^2^) with a PE of 55%. A significant correlation (r^2 ^= 0.30, *P *< 0.0001, n = 123) between CI_PPnon-cal. _and CI_TPTD _was observed after weaning from CPB (T3) with a mean bias of 0.0078 L/minute/m^2 ^(LOA: -1.69 to + 1.68 L/minute/m^2^) and an overall PE of 51%. After 45 minutes (T4), pulse power calibration was performed and CI_PPcal. _showed a significant correlation to CI_TPTD _(r^2 ^= 0.77, *P *< 0.0001, n = 59) with a mean bias of 0.0071 L/minute/m^2^, LOA from -0.89 to +0.91 L/minute/m^2 ^and an overall PE of 28%.

**Table 2:** Bland-Altman analysis showing 95% limits of agreement, confidence interval and percentage error

	T1	T2	T3	T4
	
**n**_**data**_**/n**_**patient**_	n = 84/n = 42	n = 164/n = 42	n = 123/n = 42	n = 59/n = 40
	CI_PPnon-cal._	CI_PPcal._	CI_PPnon-cal._	CI_PPcal._
**Mean (L/minute/m**^**2**^**)**	2.47	2.33	3.35	3.24
**Bias (L/minute/m**^**2**^**)**	0.36	0.075	0.0078	0.0071
**SD of bias (L/minute/m**^**2**^**)**	1.07	0.65	0.86	0.46
**CI of LOA (L/minute/m**^**2**^**)**	0.56	0.34	0.45	0.24
**95% Limits of agreement (L/minute/m**^**2**^**)**	-1.73 to +2.46	-1.19 to +1.34	-1.69 to +1.68	-0.89 to +0.91
**Percentage error (%)**	86	55	51	28

CI_PPnon-cal._, cardiac index by uncalibrated pulse power analysis; CI_PPcal._, cardiac index by calibrated pulse power analysis; CI_TPTD_, cardiac index by transpulmonary thermodilution, CI of LOA, confidence interval of the limits of agreement; Values are given as mean ± SD.

Trends of percentage changes in CI measured by pulse power analysis (ΔCI_PPnon-cal._, ΔCI_PPcal_) and transpulmonary thermodilution (ΔCI_TPTD_) are presented in detail (see Additional file 1, Figure S1). Bland-Altman analysis showed a significant correlation for ΔCI_PPnon-cal. _and ΔCI_TPTD _(r^2 ^= 0.27, *P *= 0.003) in T1 with LOA from -62 to 67%. After calibration (T2), correlation between ΔCI_PPcal. _and ΔCI_TPTD _again was statistically significant (r^2 ^= 0.30, *P *<0.0001), with LOA ranging from -42 to 36%. In time period 3 after weaning from CPB, ΔCI_PPnon-cal. _correlated with ΔCI_TPTD _(r^2 ^= 0.18, *P *= 0.01, LOA of -56 to 56%). After calibration (T4), ΔCI_PPcal. _indicated a statistically significant association (r^2 ^= 0.52, *P *= 0.005) with ΔCI_TPTD _and showed LOA from -20 to 19%. Results from ROC analysis showing the ability of ΔCI_PPnon-cal. _and ΔCI_PPcal. _to predict a ΔCI_TPTD _>15% are available (see Additional file 1, Table S1). Only ΔCI_PPcal. _was able to predict ΔCI_TPTD _>15% with a sensitivity of 90% and a specificity of 80% (AUC: 0.83, *P *= 0.03).

**Figure 3:**
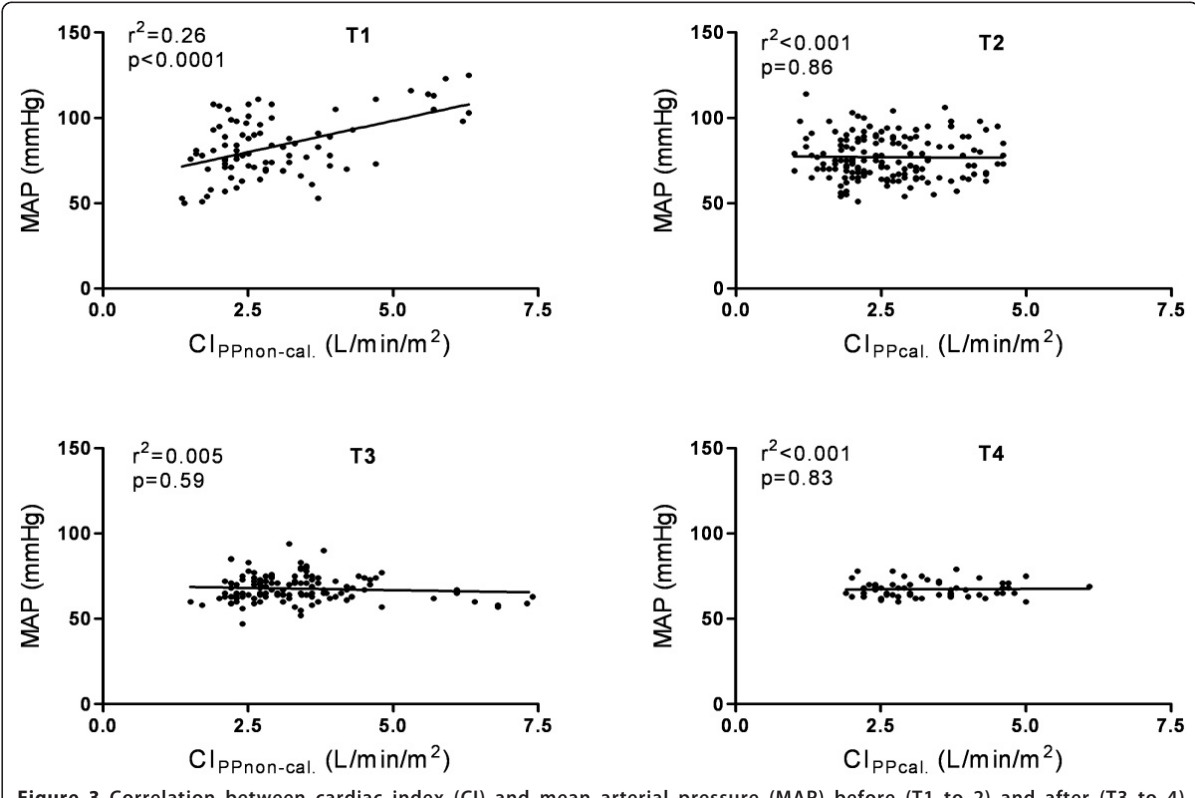
**Correlation between cardiac index (CI) and mean arterial pressure (MAP) before (T1 to 2) and after (T3 to 4) cardiopulmonary bypass**.

Correlation between MAP, CI_PPnon-cal. _and CI_PPcal., _before and after CPB is illustrated in Figure 3. Before CPB (T1), higher MAP values were significantly associated with higher CI_PPnon-cal. _(r^2 ^= 0.26, *P *<0.0001). CI_TPTD _showed no correlation with MAP before (r^2 ^< 0.01, *P *= 0.46) and after (r^2 ^= 0.03, *P *= 0.05) CPB. There was no significant relationship between CI_PPnon-cal. _and systemic vascular resistance (T1: r^2 ^= 0.004, *P *= 0.49; T2: r^2 ^= 0.02, *P *= 0.11; T3 r^2 ^= 0.02, *P *= 0.10, T4 r^2 ^= 0.01, *P *= 0.37) during the whole study period (T1 to T4).

## Discussion

The main findings of the present investigation is that CI measurement by uncalibrated arterial pulse power analysis was not able to reliably measure CI compared with TPTD before and after CPB. After calibrating the pulse power algorithm with TPTD, PE was acceptable (<30%) after CPB. In a subset of the observed patients before CPB, higher MAP values showed a significant relationship with CI_PPnon-cal._.

Arterial pulse power analysis for continuous CI measurement was introduced several years ago. Until recently, this system required a lithium indicator dilution in order to calibrate for individual aortic compliance. The new monitoring system LiDCO_*Rapid *_has been developed to provide continuous CI measurement without the need for calibration by using patient specific data for estimation of arterial compliance. To the best of our knowledge this is the first study analysing the accuracy of uncalibrated and calibrated pulse power analysis in patients undergoing coronary artery surgery.

Applying criteria proposed by Critchley and colleagues [21] to compare a new method of CI measurement with an established one, we regarded the pulse power analysis method as not interchangeable with the reference method (TPTD) if the percentage error exceeded 30%. During the first 30 minutes after induction of anaesthesia we found no correlation between CI_PPnon-cal. _and CI_TPTD _and obtained a percentage error of 86%. This value is considerably above the 30% limit of interchangeability and illustrates the difference we observed during the first period of time. To determine the influence of calibration, pulse power analysis was calibrated at defined time points before and after cardiopulmonary bypass by transpulmonary thermodilution. Accordingly, calibration should lead to an adequate accuracy and precision with respect to the reference technique, at least in the immediate period following calibration. In this context, we did not record continous cardiac output generated by the PiCCO monitoring system (PCCO), because due to our repeated calibrations we would have obtained a perfect PCCO (calibrated to the actual aortic impedance every 15 minutes by transpulmonary thermodilution), which would have induced a large bias in favor of PCCO. Several studies could demonstrate a less reliable measurement of CO by PCCO in patients undergoing cardiac surgery and in the presence of low vascular resistance after a longer period of time had elapsed after the last calibration [10,22,23].

However, though we found a significant correlation between CI_PPcal. _and CI_TPTD _(r^2 ^= 0.42, *P *< 0.0001) at T2 after pulse power calibration before CPB, PE was 55%, clearly exceeding the 30% limit mentioned before. After cardiopulmonary bypass, CI_PPnon-cal. _and CI_PPcal. _once again showed a significant correlation with CI_TPTD _and PE was 51% and 28%. As recommended by recent literature, we calculated the precision of CI_PPnon-cal./cal. _before and after CPB [24] and obtained a sufficient precision confirming our personal experience as we observed no rapid changes in CI during data recording. An explanation of these results can be found in the method underlying uncalibrated arterial pulse wave analysis. The physiological foundation of arterial pressure curves is the proportional relation of aortic pulse pressure and stroke volume and their inverse relation to aortic compliance [25,26]. Based on the windkessel model by Otto Frank arterial waveform analysis is influenced by three vascular properties: resistance, compliance and impedance [27]. However, several confounders such as individual changes in vascular compliance and resistance [28], gender [29] or vascular diseases [30] may influence this relationship in an unforeseen way. Recently, detrimental influence of significant changes of blood pressure on the accuracy of uncalibrated waveform analysis was reported both in animals and humans [25,31]. Because of the individually different relationship between changes in aortic compliance and changes in stroke volume, the increased arterial waveform could be inadvertently misinterpreted as an increase in stroke volume [32]. In accordance, we could demonstrate a significant correlation between MAP and CI_PPnon-cal. _(r^2 ^= 0.26, *P *< 0.0001) at T1, meaning that higher MAP values were associated with higher CI_PPnon-cal. _values. It must be noted, however, that this correlation is based on few data points from a small number of patients observed in T1. Additionally, the absence of correlation between MAP and CI_TPTD _emphasizes the fact that arterial compliance differed from patient to patient. As mentioned above, aortic compliance is linked to a non-linear response to arterial pressure and since the individual aortic cross sectional area is unknown, these uncertainties could lead to imprecision in determination of cardiac index by arterial waveform analysis. Therefore, this emphasizes the use of thermodilution to provide maximum accuracy during haemodynamic measurements.

Changes of systemic vascular resistance during surgery or intensive care therapy are caused by various factors such as temperature, fluid administration or decreased and increased sympathetic tone. We observed a significant lower systemic vascular resistance index (*P *< 0.05) after weaning from CPB but found no correlation between CI_TPTD_, CI_PPnon-cal./cal. _and systemic vascular resistance before and after CPB. In contrast to our findings, other observations recently reported a significant negative impact on the accuracy of arterial pulse wave analysis in patients with septic shock [33,34] and due to changes in vascular tone by vasoactive agents or intra-peritoneal hypertension [19,35]. To avoid misinterpretation in the presence of disturbing factors and to achieve the required precision, monitoring systems based on arterial waveform analysis should be able to recalculate arterial compliance at short intervals [32]. In this context, the frequency of recalculation and the underlying algorithm of uncalibrated pulse power analysis have not yet been published.

Besides the acquisition of exact CI data, the LiDCO_*Rapid *_monitoring system was also developed for evaluation and reflection of haemodynamic changes and trends during the perioperative period. In case of a critically ill patient, physicians are advised by the manufacturer to calibrate the system. Many patients undergoing elective major surgical procedures exhibit several co-morbidities, such as coronary artery disease and organ dysfunction without being in a life-threatening condition. Accordingly, with respect to this patient population most clinicians are more interested in perioperative haemodynamic changes or trends than intermittent absolute CI values. Furthermore, to avoid misleading interpretation of the Bland-Altman analysis, trends of percentage changes in CI were calculated [36] and changes of CI obtained by transpulmonary thermodilution <15% were excluded from further analysis as noise [20].

In our study, trends of percentage changes in CI measured by pulse power analysis (ΔCI_PPnon-cal./PPcal._) and transpulmonary thermodilution (ΔCI_TPTD_) revealed a weak but significant correlation before and after CPB. Calibration of pulse power analysis improved statistical significance, as well as the measurements obtained at lower MAP values immediately after CPB. We observed the best correlation of changes in CI between transpulmonary thermodilution and pulse power analysis after CPB and calibration; however, the patient sample was limited at T4 and, therefore, these data should be interpreted with caution. However, ROC analysis for prediction of ΔCI_TPTD _>15% showed that only ΔCI_PPcal. _was able to track haemodynamic changes and trends with sufficient sensitivity and specificity.

Some limitations of our study must be noted. We investigated a monitoring system developed to reflect haemodynamic trends, rather than measuring accurate CI. However, a prerequisite for using a system to guide goal-directed haemodynamic therapy in clinical settings is to understand the precision and the limitation of a monitoring technique. Furthermore, transpulmonary thermodilution implies some limitations particularly after weaning from cardiopulmonary bypass with ongoing thermal changes, leading to a higher bias caused by reduced accuracy of the reference technique [10]. However, we observed better correlation between CI and trends of CI by transpulmonary thermodilution and calibrated pulse power analysis after weaning from CPB. Due to the fact that we did not assess CI by uncalibrated and calibrated pulse power analysis at the same time but under different haemodynamic conditions, this could have induced a small bias especially in the immediate period following CPB. In this context, CI_PP _is probably also influenced by systolic arterial pressure which was unfortunately not recorded during the study period. Finally, we excluded patients with haemodynamic instability or shock and investigated patients undergoing elective coronary surgery with normal left ventricular function and without continuous application of vasoactive drugs. Therefore, our results cannot be extrapolated to patients with impaired left ventricular function, low cardiac output or patients receiving inotropic or vasoactive support.

## Conclusions

With respect to the absolute values of CI measurement, the less invasive technique of uncalibrated pulse power analysis was not interchangeable with transpulmonary thermodilution, both before and after CPB. Calibration of pulse power analysis improved accuracy, but PE was only acceptable after CPB. Correlation between MAP and CI_PPnon-cal. _in a subset of patients at T1 suggests that in the presence of high blood pressure, data from uncalibrated pulse power analysis should probably be interpreted with caution. Only calibrated pulse power analysis was able to reliably track haemodynamic changes and trends. As only a homogeneous elective patient collective was investigated, the present results, however, cannot be generalized and transferred to other groups of patients.

## Key messages

•	Uncalibrated pulse power analysis was not interchangeable with transpulmonary thermodilution before and after CPB.

•	Calibration improved accuracy, but pulse power analysis was still not consistently interchangeable with transpulmonary thermodilution.

•	Only calibrated pulse power analysis was able to track the percentage of changes in CI measured by transpulmonary thermodilution.

•	Uncalibrated pulse power analysis was significantly influenced by MAP in a subset of the observed patients, requiring further investigation in different patient populations.

## Abbreviations

CABG: coronary artery bypass grafting; Cal: calibrated; CI: cardiac index; CPB: cardiopulmonary bypass; ECG: electrocardiogram; HR: heart rate; LOA: limits of agreement; MAP: mean arterial pressure; NIBP: non-invasive blood pressure; Non-cal: uncalibrated; PAC: pulmonary artery catheter; PE: percentage error; PP: pulse power analysis; SpO_2: _peripheral oxygen saturation; TPTD: transpulmonary thermodilution.

## Competing interests

Prof. Bein is a member of the medical advisory board of Pulsion Medical Systems (Munich, Germany) and has received honoraria for consulting and giving lectures. All other authors declare that they have no competing interests.

## Authors' contributions

OB conducted the study, analyzed the data and drafted the manuscript. JR has made substantial contributions to data acquisition and has been involved in drafting the manuscript. JH helped to draft the manuscript and analyse the data. MG participated in statistical analysis and helped draft the manuscript. PM participated in study design and coordination and helped to draft the manuscript. JS participated in data analysis and coordination of the study. MS has been involved in drafting the manuscript and participated in study design. BB has been involved in drafting the manuscript, data analysis and has given final approval of the version to be published. All authors read and approved the final manuscript.

## Supplementary Material

Additional file 1 **Figure S1 and Table S1**. Figure S1: Correlation of changes in cardiac index (ΔCI). Correlation and Bland-Altman analysis of changes (%) in cardiac index (ΔCI) measured by pulse power analysis (ΔCI_PP_) and transpulmonary thermodilution (ΔCI_TPTD_) before (T1 to 2) and after (T3 to 4) cardiopulmonary bypass. Table S1: ROC-analysis to predict a change in CI by TPTD (ΔCI_TPTD_) >15%. Area under the Receiver Operating Characteristic Curve showing the ability of uncalibrated and calibrated pulse power analysis to predict a change in CI by TPTD (ΔCI_TPTD_) >15%. Click here for file
