# Supplementary material for: Uncalibrated pulse power analysis fails to reliably measure cardiac output in patients undergoing coronary artery bypass surgery
Source: Crit Care. 2011 Feb 28;15(1):R76. doi: 10.1186/cc10065 (PMC3222009; doi:10.1186/cc10065)
Supplement: Additional file 1 — Figure S1 and Table S1. Figure S1: Correlation of changes in cardiac index (ΔCI). Correlation and Bland-Altman analysis of changes (%) in cardiac index (ΔCI) measured by pulse power analysis (ΔCIPP) and transpulmonary thermodilution (ΔCITPTD) before (T1 to 2) and after (T3 to 4) cardiopulmonary bypass. Table S1: ROC-analysis to predict a change in CI by TPTD (ΔCITPTD) >15%. Area under the Receiver Operating Characteristic Curve showing the ability of uncalibrated and calibrated pulse power analysis to predict a change in CI by TPTD (ΔCITPTD) >15%. [file cc10065-S1.DOC]

**Figure S1:** Correlation and Bland-Altman analysis of changes (%) in cardiac index (∆CI) measured by pulse power analysis (∆CI_PP_) and transpulmonary thermodilution (∆CI_TPTD_) before (T1-2) and after (T3-4) cardiopulmonary bypass.

| **Table S1:** Area under the Receiver Operating Characteristic Curve showing the ability of uncalibrated and calibrated pulse power analysis to predict a change in CI by TPTD (∆CI_TPTD_) >15% | | | | | | |
| --- | --- | --- | --- | --- | --- | --- |
|  |  |  |  | | |  |
|  | AUC | 95% CI | Threshold value (%) | Sensitivity (%) | Specificity (%) | p-value |
|  |  |  |  |  |  |  |
| **∆CI_PPnon-cal_. präBP** | 0.59 | 0.39 – 0.79 | n.a. | n.a. | n.a. | 0.42 |
| **∆CI_PPcal_. präBP** | 0.53 | 0.42 – 0.65 | n.a. | n.a. | n.a. | 0.54 |
| **∆CI_PPnon-cal_. postBP** | 0.59 | 0.47 – 0.72 | n.a. | n.a. | n.a. | 0.20 |
| **∆CI_PPcal_. postBP** | 0.83 | 0.59 – 1.10 | ≥8.5 | 90 | 80 | 0.03 |

∆CI_PPnon-cal._, changes in cardiac index by uncalibrated pulse power analysis; ∆CI_PPcal._, changes in cardiac index by calibrated pulse power analysis; AUC, area under the curve; n.a., not assessed.
